# The non-clonality of drug resistance in Beijing-genotype isolates of *Mycobacterium tuberculosis *from the Western Cape of South Africa

**DOI:** 10.1186/1471-2164-11-670

**Published:** 2010-11-26

**Authors:** Thomas R Ioerger, Yicheng Feng, Xiaohua Chen, Karen M Dobos, Thomas C Victor, Elizabeth M Streicher, Robin M Warren, Nicolaas C Gey van Pittius, Paul D Van Helden, James C Sacchettini

**Affiliations:** 1 Department of Computer Science and Engineering, Texas A&M University, College Station, TX USA; 2 Department of Biochemistry and Biophysics, Texas A&M University, College Station, TX USA; 3 Department of Microbiology, Immunology, and Pathology, Colorado State University, Fort Collins, CO USA; 4 DST/NRF Centre of Excellence in Biomedical Tuberculosis Research, US/MRC Centre for Molecular and Cellular Biology, Division of Molecular Biology and Human Genetics, Department of Biomedical Sciences, Faculty of Health Sciences, Stellenbosch University, P.O. Box 19063, Tygerberg 7505, South Africa

## Abstract

**Background:**

The Beijing genotype of *M. tuberculosis *is a virulent strain that is disseminating worldwide and has a strong association with drug resistance. In the Western Cape of South Africa, epidemiological studies have identified the R220 cluster of the Beijing genotype as a major contributor to a recent outbreak of drug-resistant tuberculosis. Although the outbreak is considered to be due to clonal transmission, the relationship among drug resistant isolates has not yet been established.

**Results:**

To better understand the evolution of drug resistance among these strains, 14 drug-resistant clinical isolates of the Beijing genotype were sequenced by whole-genome sequencing, including eight from R220 and six from a more ancestral Beijing cluster, R86, for comparison. While each cluster shares a distinct resistance mutation for isoniazid, mapping of other drug-resistance mutations onto a phylogenetic tree constructed from single nucleotide polymorphisms shows that resistance mutations to many drugs have arisen multiple times independently within each cluster of isolates. Thus, drug resistance among these isolates appears to be acquired, not clonally derived. This observation suggests that, although the Beijing genotype as a whole might have selective advantages enabling its rapid dissemination, the XDR isolates are relatively less fit and do not propagate well. Although it has been hypothesized that the increased frequency of drug resistance in some Beijing lineages might be caused by a mutator phenotype, no significant shift in synonymous substitution patterns is observed in the genomes.

**Conclusion:**

While MDR-TB is spreading by transmission in the Western Cape, our data suggests that further drug resistance (i.e. XDR-TB) at this stage is acquired.

## Background

The Beijing genotype of *Mycobacterium tuberculosis *is a virulent strain that originated out of East Asia [1] and has disseminated around the world [2,3]. It is a member of principle genetic group 1 [4], bearing the markers KatG 463Leu and GyrA 95Thr, and is characterized by a spoligotype 000000000003771. Isolates of the Beijing strain have been associated with decreased survival times in mice [5-7] and in the rabbit meningitis model [8], and increased growth rates in human macrophages [9,10]. Some patients infected with Beijing genotype show increased radiographic cavitation [11,12] and experience more treatment failures, independent of differences in drug resistance [13]. One potential explanation for the increased virulence is the production of phenolglycolipid (PGL), a surface antigen that suppresses the Th1 response [14]. PGL is produced in Beijing strains, but is not produced by members of the other principle genetic groups (2 and 3), such as *M. tuberculosis *H37Rv, because the polyketide synthase pks15/1 has a frameshift mutation splitting it into two separate open reading frames (ORFs) [15].

The Beijing genotype is strongly associated with drug resistance [16], including multi-drug resistance (MDR-TB) and extensive-drug resistance (XDR-TB). One of the first outbreaks of MDR-TB, which occurred in New York City in the early 1990's, was found to be a clonal expansion of a variant of the W-Beijing strain [17,18]. The Beijing strain was also associated with an outbreak in Azerbaijan prisons, where nearly all TB infections were Beijing, and >50% were MDR [19]. The Beijing genotype has been reported to account for 34% of the XDR cases across South Africa [20]. Similar findings of increased association of drug resistance with strains of the Beijing genotype have been reported in India [21], Russia [22], Korea [23], Vietnam [24], Japan [25], and Germany [26]. In a large-scale study that included both civilians and prison inmates in Russia, 67% of the TB infections were Beijing, but the frequency of resistance to drugs like isoniazid, rifampicin, streptomycin, and ethambutol was nearly twice as high among Beijing as non-Beijing isolates [11]. Although these studies were performed in different populations using varying methodologies, taken together, they support the general view that infections with the Beijing strain are more likely to be drug resistant than other strains of TB.

Currently, the Beijing strain constitutes a significant component of a major outbreak of TB in the Western Cape of South Africa, where it represents 36.5% of the drug-resistant cases (in a sample between 2005 and 2006 [27]). The proportion of the Beijing genotype among drug-resistant cases is inflated relative to the overall proportion of Beijing strains among drug-susceptible TB cases in the region, which was estimated at 21.9% in Cape Town (between 1993 and 2004) [28]. In another study of TB cases in the Western Cape region between 2001 and 2002, 28% of drug-resistant cases were of the Beijing genotype, whereas 17% of cases overall were Beijing [29]. Epidemiological studies suggest that the Beijing genotype is highly transmissible (based on geographical clustering of individual strains within households and communities [30]), leading to a hypothesis that drug resistance is spreading clonally through the region. However, the clonal expansion hypothesis contrasts with most TB outbreaks, which are often found to be constituted of a mixture of genotypes [31]. In fact, drug resistant mutations often incur a relative fitness cost, making it more difficult for them to compete [32]. van der Spuy et al. [28] found that the increase in Beijing strains in Cape Town was primarily due to drug-susceptible strains. Over a 12-year period, the number of drug-susceptible Beijing isolates increased exponentially with a doubling time of ~4 years, whereas the frequency of most other genotypes, including drug-resistant Beijing isolates, remained relatively constant. This observation was taken to suggest that the success of the Beijing strain is due overall to increased virulence rather than transmissibility [28], and that drug-resistant strains of Beijing were less fit that drug-susceptible Beijing strains. Thus the association of Beijing with drug-resistance could be a side-effect due to the increased overall success of this strain.

The Beijing genotype family can be divided into seven lineages, progressing from "ancestral" (sublineage 1) to "modern" (sublineage 7). These distinctions are based on comparison of IS6110 insertion sites, regions of deletion, and other markers [33]. Sublineage 7 is most prevalent in the Western Cape (72.6% in Cape Town between 1993 and 2004), which, discounting founder effects, is interpreted to mean that it has higher fitness [33]. Some studies have suggested that different lineages have different propensities to develop drug resistance. For example, Mokrousov et al. [34] found that ancestral lineages in China had a higher frequency of resistance to rifampicin and pyrazinamide. However, these findings were contradicted by a larger study of Beijing isolates in South Africa that found no statistically significant difference in drug resistance among the seven lineages [33].

One particular sub-group of the Beijing family has recently been identified, cluster R220 (based on IS6110 RFLP banding patterns), that is overrepresented among drug-resistant isolates in the Western Cape of South Africa [27]. R220 is a member of sublineage 6, representing a modern Beijing variant. R220 constituted over 75% of isolates of the Beijing clade in 2005-2006 in the Western Cape, and accounted for 42% of the increase in drug-resistant cases since 2001 [27]. R220 was also found to be prevalent among children infected with drug-resistant TB in the region [35]. Cluster R220 strains share the same c-15t *inhA *promoter mutation responsible for resistance to isoniazid and many isolates (nearly 75%) also have the Ser531Leu (TTG) *rpoB *conferring rifampicin resistance, suggesting that it has evolved into a distinct MDR clone. If it were a distinct clone, this might imply that it has acquired compensatory mutations that enable it to tolerate the fitness cost associated with drug resistance mutations or enhance its transmissibility in the population.

Although R220 is a well-defined cluster in terms of spoligotyping and IS6110 RFLP fingerprinting, these are still coarse-grained methods of genotyping and do not guarantee that the drug-resistant isolates in this cluster are clonally derived. In order to get a more fine-grained picture of the R220 cluster, we performed whole-genome sequencing on eight drug-resistant isolates of R220 from various locations in the Western Cape of South Africa. For comparison, we also sequenced HN878, a drug-susceptible isolate from the US, and six drug-resistant isolates of the R86 cluster, which is part of the more ancestral sublineage 1. Using these whole-genome sequences, we reconstruct a phylogenetic tree and map drug-resistance mutations on them to examine the hypothesis of clonality.

## Methods

### Selection of Strains for Sequencing

**Figure 1:**
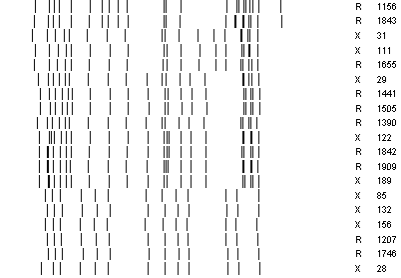
**IS*6110 *RFLP fingerprints of the Beijing clinical isolates sequenced in this study**.

*M. tuberculosis *HN878 was obtained from the NIH TB Vaccine Testing and Research Materials Laboratory at Colorado State University. Fourteen additional drug-resistant strains of the Beijing genotype were selected for whole-genome sequencing and comparative analysis. The isolates were sampled from a database maintained at the DST/NRF Centre, Stellenbosch University, representing drug resistant TB cases from a variety of hospitals in the Western Cape, South Africa (see Table 1). The samples were all selected to be isoniazid-resistant, and were chosen to span a range of degrees of drug resistance, including cases that are mono-resistant (isoniazid), MDR (isoniazid and rifampicin), pre-XDR (MDR plus resistance to either a fluoroquinolone or an aminoglycoside), and XDR. No other biases (e.g. age, gender, HIV status) were applied in the sampling. All 14 isolates have the Beijing spoligotype, 00000000003771. Six of the isolates investigated in this study are from sublineage 1, representing the atypical Beijing genotype (ancestral, family F31). IS*6110*-RFLP analyses have classified these strains as being members of cluster R86. The remaining 8 isolates are from cluster R220 in sublineage 6, which represents "typical" Beijing genotype strains (more recently evolved). The IS*6110 *RFLP patterns used for determining cluster membership of these isolates are shown in Figure 1.

**Table 1:** Beijing strain clinical isolates from South Africa subjected to whole-genome sequencing.

Strain ID	Origin (hospital)	Sub-lineage	Family	Cluster	DR type	Combined resistance profiles determined by DST	Combined sensitivity profiles determined by DST
**R1207**	George	1	F31	R86	MDR	INH, RIF	EMB
**X132**	Khayelitsha	1	F31	R86	pre-XDR	INH,RIF,AMI,CAP,STR,KAN	ETH,OFL,EMB
**X28**	Dysselsdorp	1	F31	R86	XDR	INH,RIF,AMI,CAP,OFL,STR, KAN	ETH, EMB
**R1746**	Mossel Bay	1	F31	R86	MDR	INH, RIF	EMB
**X156**	Brooklyn Chest	1	F31	R86	pre-XDR	INH,RIF, AMI,CAP,STR,KAN	ETH,OFL,EMB
**X85**	George	1	F31	R86	XDR	INH,RIF, AMI,OFL,KAN,ETH	CAP,EMB
**R1909**	Worcester	6	F29	R220	MDR	INH,RIF,EMB	
**R1842**	George	6	F29	R220	Mono	INH	RIF, EMB
**X122**	Huguenot	6	F29	R220	pre-XDR	INH,RIF,OFL	ETH,AMI,EMB
**R1390**	Stellenbosch	6	F29	R220	Mono	INH	RIF,EMB
**X189**	Victoria Hos.	6	F29	R220	XDR	INH,RIF,AMI,CAP, OFL,KAN,	ETH,STR
**R1505**	George	6	F29	R220	MDR	INH, RIF	EMB
**R1441**	George	6	F29	R220	Mono	INH	RIF,EMB
**X29**	Retreat	6	F29	R220	pre-XDR	INH,RIF,AMI,STR,KAN	EMB,OFL,CAP,ETH

INH = isoniazid RIF = rifampicin STR = streptomycin EMB = ethambutol ETH = ethionamide AMI = amikacin KAN = kanamycin CAP = capreomycin OFL = ofloxacin

### Drug-Susceptibility Testing

Drug susceptibility testing for isoniazid (0.2 μg/ml), rifampin (1 μg/ml), ethambutol (7.5 μg/ml), ethionamide (20 μg/ml), amikacin (30 μg/ml) and ofloxacin (2 μg/ml) was performed using the indirect proportion method on Middlebrook's medium [36]. Additional drug susceptibility testing was done using the proportion method on Middelbrook's 7H10 medium containing ofloxacin, amikacin, kanamycin and capreomycin at the critical concentration of 2 μg/ml, 5 μg/ml, 5 μg/ml and 10 μg/ml, respectively.

### DNA Preparation and Sequencing Reaction

Sequencing of the genomes of 15 Beijing strains of *M. tuberculosis *was carried out on an Illumina Genome Analyzer II (Illumina, Inc.). In this study, Illumina Paired-End sequencing method (PE) was used. The cetyltrimethylammonium bromide (CTAB)-lysozyme method was used for extraction and purification of genomic DNA [37]. Suitable genomic DNA samples for the GAII were prepared as described on the sample preparation protocol (Illumina). 2-3 μg of genomic DNA was used for sample preparation. Genomic DNA was sheared by a nebulizer to generate DNA fragments for the Illumina Paried-End Sequencing method. The specific oligonucleotides (Illumina adapters) designed for PE sequencing were ligated to both ends of DNA fragments with the TA cloning method. Adapter-ligated DNA fragments of length 350-400 bp were isolated from a 2% agarose gel (Certified low-range Ultra Agarose, BIO-RAD) by using QIAquick Gel Extraction Kit (QIAGEN). Then the fragments were amplified by PCR reaction to generate the DNA library (15-30 ng/μl). The median size of the library was estimated by examining the 2% agarose gel image. The molarity of the DNA library was estimated as described in the sample preparation protocol (Illumina). The DNA libraries (5 picomoles each), including the ϕX174 control (bacteriophage DNA), were loaded on the flow cell for the cluster generation and sequencing. 79 cycles of images were collected, representing two 36-bp reads (paired ends) plus 7 bases representing id tags for multiplexing. The images were analyzed using version 1.4 of the GAPipeline software supplied by Illumina, producing files with 10-20 million pairs of 36 bp reads for each genome.

### Sequence Determination and Bioinformatics

The reads were analyzed by comparative genome assembly to determine the complete sequence of each genome using custom software developed in our lab. The reads were first mapped (aligned) against the genome of H37Rv as a reference sequence. The mapping of each read was accomplished by ungapped alignment to the reference genome (including the reverse complement strand), allowing at most two mismatches out of 36 nucleotides. Initially, the reads that were pair-mates were mapped independently; subsequently, locations of reads for which the paired-end did not match within 300 bp were discarded. The mapped reads were used to assemble a list of the nucleotides observed at each position within the reference genome contributed by all the reads that overlapped it. Base calls were made by a maximum likelihood calculation, computed as the product of the probabilities for each base at each position using uncertainties estimated during image analysis. Sites where apparent differences were observed were subjected to local contig-building, in order to determine whether the difference was due to a nucleotide substitution or a small insertion or deletion.

For each genome, a list of verified differences was prepared and used to modify the reference genome to produce an intermediate ('edited') genome. Then the process was repeated by re-mapping the reads against the edited genome, and re-calling nucleotides at each position. For any sites that still had zero coverage, the base from the reference strain was used. This primarily included regions with exceptionally high GC-content (80-90%).

Large-scale deletions were identified by analyzing paired-end data for reads whose paired-end maps an unusually long distance away (i.e. >300 bp, compared to average read length of ~200). Large-scale insertions were determined by building contigs that spanned fragments, neither of whose paired ends mapped into the H37Rv genome. These ~200 bp fragments were then assembled into larger contigs using Newbler (software from Roche, inc.), localized in the genome using paired-end information, and identified by performing BLAST searches on the NCBI website.

Virtual spoligotyping was performed by aligning (without gaps) all the reads obtained for each strain against each of the 43 spacer sequences (26-bp oligos) from the direct repeats (DR) regions [38]. The number of matching reads for each spacer was counted, considering both forward and reverse-complement sequences, and accepting up to 1 nucleotide mismatch. Spacers with 0 matches were interpreted as missing.

Whole-genome alignments of the Beijing sequences to other mycobacterial strains were generated using MUMMER version 3.20 [39] to identify maximal stretches of perfectly matching regions, selecting optimal order-preserving assignments of matches between the genomes based on the longest increasing subsequence algorithm [40], and then using in-house sequence-alignment tools to determine alignments of the intervening regions with mismatches and/or gaps.

The complete genome sequences for *M. tuberculosis *strains HN878, R1207 (representative of R86 cluster), and X122 (representative of R220 cluster) have been deposited in GenBank with accession numbers ADNF01000000, ADNH01000000, and ADNG01000000, respectively.

## Results

### Whole-Genome Sequencing

In order to establish a complete genome sequence to use as a reference for sequencing of other members of the Beijing strain family, HN878 was chosen as a representative strain and the genome was sequenced using Solexa sequencing technology. HN878 is a fully drug-susceptible member of the modern (typical) W-Beijing family which was isolated in Houston, TX as part of a TB outbreak in the 1990's, and has been used as a reference strain in multiple studies to characterize virulence and other properties of the Beijing strain [5,7,8]. HN878 was sequenced in paired-end mode using 36 bp reads. Mapping of reads to unique spacer sequences in the direct-repeats (DR) region confirms that HN878 has the characteristic 000000000003771 spoligotype associated with the Beijing strain family. The genome sequence of *M. tuberculosis *H37Rv (NCBI accession: NC_000962) was used as a reference sequence for comparative assembly of the HN878 genome. The mean depth of coverage was 70.8x, and 98.4% of the genome was covered by at least one read. The remaining uncovered regions are primarily associated with the family of PGRS genes, and are likely due to inefficient sequencing of GC-rich regions. In HN878, 91.5% of sites with zero coverage (60,454/66,065) were located in PGRS genes.

Among 1546 SNPs relative to H37Rv, 1296 occur in protein-coding regions. Only 1063 of 3989 genes contain a polymorphism of any type, and the remaining 73% of the genes in the genome are identical with the sequence in H37Rv. 75 genes contain frameshift mutations (see Additional file 1, Table S1). No mutations typically associated with drug resistance were found in the following genes, consistent with the pan-susceptible phenotype of this clinical isolate: *inhA, katG, ethA, gyrA, iniABC, kasA, ndh, rpoB, rpsL, rrs, pncA*, and *embB*. HN878 has the *katG *R643L and *gyrA *S95T alleles, confirming its membership in principle genetic group 1 [4]. Further details on SNPs and indels in HN878 (Table S2) relative to H37Rv, including IS6110 transpositions (Table S3) are described in the Additional file 1.

Then the genomes of the 14 Beijing clinical isolates from South Africa were sequenced in paired-end mode, using HN878 as a reference sequence (Table 2). The coverage ranged between 29x and 98x, and the completion was 98.5-99.4%.

**Table 2:** Sequencing statistics on Beijing genotype strains from South Africa.

strain ID	Cluster	completion	coverage	SNPs*	indels*
R1207	R86	98.77%	66.9x	689	58
X132	R86	98.71%	40.3x	671	61
X28	R86	98.35%	57.6x	681	51
R1746	R86	98.26%	50.8x	665	49
X156	R86	98.69%	106.4x	799	57
X85	R86	98.78%	97.7x	714	60
R1909	R220	98.94%	32.8x	297	54
R1842	R220	99.15%	58.2x	324	49
X122	R220	98.60%	64.1x	299	41
R1390	R220	98.50%	31.3x	273	44
X189	R220	98.85%	87.6x	392	42
R1505	R220	98.55%	29.0x	267	39
R1441	R220	99.03%	82.7x	295	35
X29	R220	99.17%	82.0x	315	41

*The number of SNPs and indels are assessed relative to HN878.

**Figure 2:**
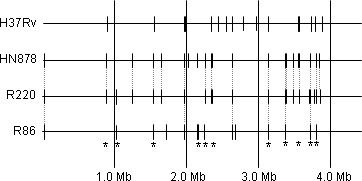
**Positions of IS*6110 *insertion sites**. Dashed lines indicate identical sites. Asterisks indicate sites where there are two or more close but distinct insertions separated by 27-663 bp.

There was a high concordance of IS*6110 *insertion sites between R220 strains and HN878 (see Figure 2). Of the 21 insertion sites in HN878, four are absent in R220, although an additional four novel insertion sites are present in this cluster (see Additional file 1, Table S4). Similar to HN878, R220 has a single insertion in the NTF region, grouping it with HN878 in the modern sublineage of Beijing strains [34]. The R220 strains lack the RD150 deletion characteristic of sublineage 7 [33,41], having Rv1671-Rv1674 intact and placing them in sublineage 6. The six R86 strains were all found to have the Beijing spoligotype and a common set of 14 IS*6110 *insertion sites (Additional file 1, Table S5), including one insertion in the *dnaA-dnaN *region. However, only 6 of these sites were shared with HN878. The R86 strains do not have any insertion in the NTF region (~3.48 Mb), classifying them as "ancestral" or "atypical" Beijing lineages [34].

Table 2 shows that the number of SNPs and indels was lower between the R220 strains and HN878 compared to the R86 strains, suggesting they are more closely related to HN878. The number of SNPs and indels compared to HN878 were 665-799 and 49-61, respectively, for the cluster R86 strains, and 267-392 and 35-54, respectively, for the cluster R220 strains.

The clinical isolates in the R220 cluster are fairly homogeneous at the genomic level. Among 1573 polymorphic sites between the R220 strains and H37Rv (SNPs not involving PPE genes, PGRS genes, or repetitive elements), 1234 differences (78.4%) were shared among HN878 and all 8 R220 strains. Some of these differences from H37Rv might be shared with other strain families like LAM. There were 113 sites (7.2%) where the R220 strains shared a difference from H37Rv but HN878 did not, and there were 91 sites (5.8%) where HN878 differed from H37Rv but the R220 strains did not. Each of the strains except one had a small number of unique SNPs (1-12).

R86 strains are found to have a set of SNPs compared to H37Rv that only partially overlaps with the SNPs in HN878. Out of a selected set of 1885 SNPs, 1126 (59.7%) were found to be common among HN878 and all six R86 strains relative to H37Rv, HN878 has 297 (15.8%) unique SNPs, and the R86 strains have 256 (13.6%) shared SNPs not in HN878. Thus, while both HN878 and the R86 share many polymorphisms presumably acquired in a common ancestor of the Beijing family, they have each diverged significantly, reflecting the difference in their lineages (sublineage 1 for R86 versus sublineage 6 for HN878).

**Figure 3:**
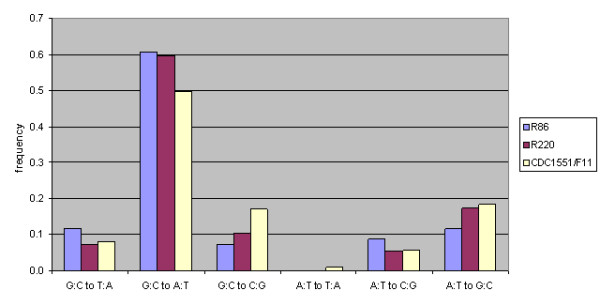
**Substitutions at synonymous sites categorized by specific base-pair replacements, showing a similar preference for G:C to A:T transversions among isolates of the R86 cluster and R220 cluster as for other mycobacterial strains such as CDC1551 and F11**. All substitutions were based on comparison to H37Rv. The total number of synonymous SNPs analyzed was 112, 126, and 537 for R86, R220, and CDC1551/F11, respectively.

When SNPs at synonymous sites are categorized into specific base-pair substitutions, the majority of replacements (~60%) are found to be G:C to A:T transversions (68/112 in the R86 cluster, and 75/126 in the R220 cluster) (Figure 3). However, a similar bias is observed among synonymous SNPs in CDC1551 or F11 (186/374 combined) when compared to H37Rv (χ^2 ^= 7.36 < 11.07 for the distribution of SNPs in the R220 cluster versus those in CDC1551/F11, which means the difference in substitution patterns is not significant at the *p *= 0.05 confidence level, *df *= 5), suggesting that the effect is not due to a Beijing-specific shift in the types of mutation.

### Drug-Resistance Mutations

Among the Beijing clinical isolates from South Africa, well-known polymorphisms are observed that correlate with drug resistance patterns. Drug-resistance polymorphisms for the 14 clinical isolates are shown in Table 3. All of the Beijing isolates from the Western Cape, South Africa included in this study are isoniazid-resistant, whereas HN878 is susceptible. All six R86 strains have the S315T mutation in *katG*, explaining the isoniazid resistance, and suggesting it was acquired prior to divergence of this cluster. X156 and X85 also have the c-15t *inhA *promoter mutation, and R1207, X132, and X28 have c-17t in the *inhA *promoter. *inhA *promoter mutations are generally considered to confer a lower-level of resistance to isoniazid (2-4 fold) as well as high-level cross-resistance to ethionamide [42]. All 6 strains also have the mutation A381P in *ethA*, a prodrug-activator, which has been suggested to also confer high-level resistance to ethionamide [43]. The polymorphisms in the R220 genome sequences are also largely consistent with the reported drug-susceptibility profiles, with the exception of ethionamide sensitivity in isolates X122, X189, and X29 (Table 4). This may relate to anomalies in drug susceptibility testing, as all R220 isolates have the c-15t *inhA *promoter mutation, which should cause ethionamide resistance [43]. In contrast to the cluster R86 isolates, the R220 isolates show no mutations in *katG*. Strains R1909 and X189 also have mutation Ile194Thr in the coding region of *inhA*, which occurs in the active site of the enoyl-ACP reductase and interferes with binding of INH-NAD and ETH-NAD adducts [44,45].

**Table 3:** Drug resistance mutations among R86 cluster isolates.

strain	**Resist**.	**sens**.	*katG*	*inhA*	*rpoB*	*embB*	*pncA*	*rrs*	*ethA*	*gyrA*
R1207	IR	B	S315T	g-17t	D435V	M306I	+c L172	a514c, a1401g	A381P	D94H
X132	IRACSK	EOB	S315T	g-17t	D435V	M306I	+c L172	a514c, a1401g	A381P	wt
X28	IRACOSK	EB	S315T	g-17t	D435V	M306I	+c L172	a514c, a1401g	A381P	D94N
R1746	IR	B	S315T	wt	S450L	M306I	C14R	a514c	A381P	wt
X156	IRACSK	EOB	S315T	c-15t	S450L	M306I	C14R	a514c, a1401g	A381P	wt
X85	IRAOKE	CB	S315T	c-15t	S450L	M306I	C14R	a514c, a1401g	A381P	D94G

wt = wild-type, I = isoniazid, R = rifampicin, S = streptomycin, A = amikacin, C = capreomycin, K = kanamycin, E = ethionamide, B = ethambutol, O = ofloxacin.

**Table 4:** Drug resistance mutations among R220 cluster isolates.

strain	**resist**.	**sens**.	*katG*	*inhA*	*rpoB*	*embB*	*pncA*	*rrs*	*rpsL*	*gyrA*
R1909	IRB		wt	c-15t, I194T	S450L	M306V	D8N	a1401g	wt	wt
R1842	I	RB	wt	c-15t	S450L	M306V	wt	c517t	wt	D94A
X122	IRO	EAB	wt	c-15t	S450L	M306I	Y103*	wt	K43R	D94G
R1390	I		wt	c-15t	wt	wt	wt	wt	wt	wt
X189	IRACOK	ES	wt	c-15t, I194T	S450L	M306V	881 bp del	a1401g	wt	D94A
R1505	IR	B	wt	c-15t	H445Y	wt	wt	wt	wt	wt
R1441	I	RB	wt	c-15t	wt	M306I	wt	wt	wt	wt
X29	IRASK	BOCE	wt	c-15t	wt	M306V	8 bp del	a1401g	wt	wt

wt = wild-type, I = isoniazid, R = rifampicin, S = streptomycin, A = amikacin, C = capreomycin, K = kanamycin, E = ethionamide, B = ethambutol, O = ofloxacin.

Rifampicin resistance is explained in three R86 strains (R1207, X132, and X28) by the D435V mutation in *rpoB*, while the other three R86 strains (R1746, X156, and X85) have S450L (equivalent to amino acids 516 and 531 in the conventional *rpoB *numbering based on *E. coli*). Both mutations are known to confer resistance to rifampicin [46]. S450L (TCG->TTG) is the most frequently observed mutation in cultures selected on rifampicin *in vitro*, implying lowest fitness cost [32]. D435V (GAC->GTC) is much less common *in vitro *(~1%) [47], but is frequently observed clinically [46,47]. Four of the five R220 isolates that are rifampicin-resistant (R1909, X122, X189, and R1505) have a mutation in *rpoB *(either S450L or H445Y). However, isolate X29 (rifampicin-resistant) does not have any mutations in *rpoB*, and conversely, isolate R1842 (rifampicin-sensitive) has S450L in *rpoB*, which in turn may reflect laboratory error. The H445Y mutation is less frequent among clinical isolates, but also occurs in the rifampicin binding site in RNA polymerase.

Resistance to aminoglycosides can result from a variety of polymorphisms, generally related to the small subunit of the ribosome. Mutations that confer resistance to streptomycin and similar compounds are usually found in the 500-bp or 900-bp region of *rrs*, the 16S rRNA, or in *rpsL*, one of the ribosomal proteins [48]. All six R86 strains have the a514c mutation in *rrs*, conferring streptomycin resistance. X28 and X156 showed streptomycin resistance in drug-susceptibility testing; data on the other four strains was not available. Kanamycin binds a different site on the ribosome and shows cross-resistance with amikacin and capreomycin [49]. The most common mutation associated with kanamycin resistance is a1401g in *rrs *[50]. All R86 strains except isolate R1746 have the a1401g mutation in *rrs*. Four of the six strains were kanamycin-resistant (the other two were not tested). The capreomycin sensitivity of isolate X85 is inconsistent with the kanamycin/amikacin resistance, and might be an anomaly in drug-susceptibility testing. In the R220 cluster, isolates X189 and X29 are resistant to kanamycin and amikacin, and both strains show the expected a1401g mutation in *rrs*, whereas X122 (amikacin-sensitive) does not. R1842 has *rrs *c517t and X122 has K43R in ribosomal protein *rpsL*, each of which should confer streptomycin resistance, though this was not tested.

Resistance to fluoroquinolones is typically caused by mutations in *gyrA *[51]. Isolates X28 and X85 were reported to be ofloxacin-resistant, and X132 and X156 were reported to be ofloxacin-sensitive (data for R1207 and R1746 was not available). Three different amino acid mutations were observed in *gyrA *among these six strains: D94H in isolate R1207, D94N in X28, and D94G in X85. The SNPs in isolates X28 and X85 explain their ofloxacin resistance, and the fact that X132 and X156 have wild-type *gyrA *sequences is consistent with their ofloxacin sensitivity. The ofloxacin resistance of isolates X122 and X189 can be explained by mutations in D94 in *gyrA. *Isolate X29, which is sensitive to oflaxacin, has a wild-type *gyrA *sequence.

All six R86 strains have the mutation M306I in *embB*, commonly associated with resistance to ethambutol [52], although all six strains were reported to be sensitive to ethambutol by drug-susceptibility testing. It is well known that drug-susceptibility testing under-reports ethambutol resistance [53]. Note that, while all 6 strains have a mutation from Met to Ile, they use different codons; 'ATA' (for R1207, X132, and X28) and 'ATC' (for R1746, X156, and X85). Many of the R220 strains have a mutation in Met306 in *embB*, either to Val or Ile, although most of the strains tested sensitive to ethambutol as well.

Although resistance to pyrazinamide was not tested, 10 of the 14 strains showed mutations in *pncA *(pyrazinamidase). One R86 isolate (X29) has a frameshift mutation of 8 bp in *pncA*, while another (X189) had a large deletion of 881 bp knocking out the entire coding region, along with the adjacent gene, Rv2044c. Strain R1909 has mutation D8N, and strain X122 has Y103* (truncation mutation). All six R220 strains show mutations in *pncA*: three strains have a frameshift mutation (+c in codon 172), and three have amino acid substitution (C14R). Multiple mutations, including frameshifts, throughout *pncA *are associated with resistance to pyrazinamide [54-56].

### Non-clonal Acquisition of Drug-Resistance Mutations

**Figure 4:**
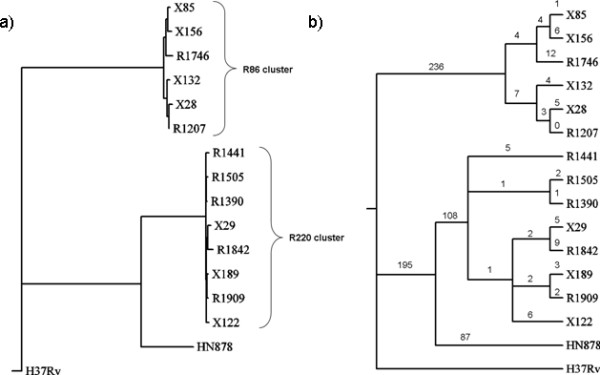
Phylogenetic tree constructed from 727 SNPs (excluding those related to drug resistance) by maximum parsimony (a), and also displayed as a cladogram (b) showing the number of changes (unique SNPs) associated with each branch.

In order to better understand the origins of drug resistance among the Beijing strains, a phylogenetic tree was constructed to identify their evolutionary relationships. Subsequently, drug resistance mutations were mapped onto the tree to determine whether the patterns of drug resistance (associations between strains) could be explained by the same topology (i.e. inheritance). A master set of 727 polymorphic sites (SNPs only) with good depth of coverage (≥10x, i.e. sites covered by at least 10 reads) across all sequenced strains was selected. A subset of 704 sites was produced by removing those involved in drug resistance (e.g. *inhA, katG, gyrA*, *rpoB*, *pncA, rrs, embB*). The 704 sites were used to construct a maximum parsimony tree using *dnapars *in PHYLIP 3.66 [57]. Figure 4 shows the phylogenetic relationship with representative branch lengths, clearly showing the tight clustering of the two groups, cluster R86 (X85, X156, R1746, X132, X28, R1207) and the R220 cluster (R1441, R1505, R1390, X29, R1842, X189, R1909, X122), indicating that both groups have evolved independently from a common ancestor.

The number of SNPs differentiating the strains within each cluster is small but non-zero. Since the relationships are difficult to see in Figure 4a, the phylogeny was re-drawn as a cladogram (without meaningful branch lengths) in Figure 4b, and each branch is labeled with the number of SNPs associated with it. The observation that cluster R86 isolates share 236 SNPs while the R220 isolates share 303 unique SNPs suggests that these two clusters are largely clonal, and that isolates within the clusters are differentiated from one another by a limited number of SNPs. For example, the similarity between X29 and R1842 is supported by two unique SNPs: Q219P in Rv2571c and V16V in *proW*. The SNPs associated with each branch point are listed in Additional file 1, Table S7. It is also interesting to note that some individual isolates have continued to diverge, for example isolate R1746 has accumulated 12 unique polymorphisms. These differentiating SNPs likely represent recent evolutionary events. According to this phylogeny, HN878 is more closely related to the R220 cluster (sharing 195 SNPs, compared to 0 shared with the R86 cluster, using H37Rv as the outgroup), but is distinguished from the R220 members by a further 195 unique differences (108 + 87). It is important to note that these polymorphisms do not necessarily imply anything about the overall population structure, due to the non-random nature of the sample. Nonetheless, they represent the diversity and inter-relationships among the 14 clinical isolates sequenced.

**Figure 5:**
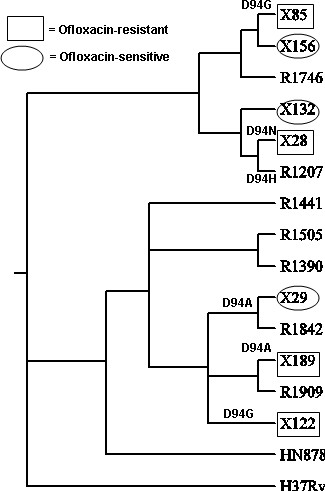
**Mutations in *gyrA *related to fluoroquinolone resistance**. Ofloxacin-resistant strains are boxed; Ofl-sensitive strains are encircled; other strains were not tested by DST.

When mutations related to drug resistance were projected onto this phylogeny, the patterns were frequently found to disagree with the phylogeny above. The most parsimonious explanation for this observation is that mutations conferring resistance to a given drug have arisen multiple times independently within both the R220 and R86 clusters. Figure 5 shows the pattern of mutations in *gyrA *conferring fluoroquinolone resistance. In these 14 Beijing strains, mutations in Asp94 appear to have arisen 6 times independently, each associated with a unique isolate (i.e. they were not clustered in the cladogram). In fact, Asp94 is mutated to 4 different amino acids (Gly: X85, X122, Asn: X28, His: R1207, Ala: X29, R1842, X189), again supporting the uniqueness of these events.

**Figure 6:**
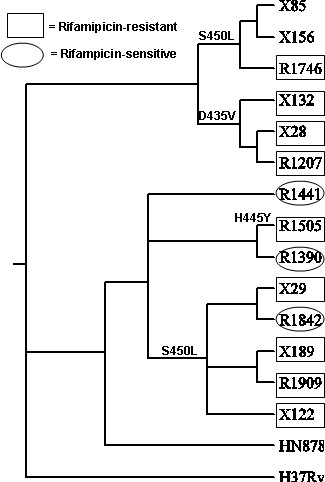
**Mutations in *rpoB *related to rifampicin resistance**.

Mutations in *rpoB *related to rifampicin resistance are shown in Figure 6. In the R86 cluster, the six isolates divide into two groups: three isolates with the S450L mutation, and three with the D435V mutation. Similarly, the S450L mutation also explains the rifampicin resistance in a group of five isolates of the R220 cluster, clearly demonstrating that each cluster has acquired rifampicin resistance independently. It is important to note that three of the eight R220 isolates did not bear the S450L mutation. Although many R220 isolates have the S450L polymorphism [58], as of 2006 only 73% of R220 isolates were RIF-resistant [27], indicating that this polymorphism had not yet achieved fixation in the population, and thus the population structure has not yet been taken over by a distinct MDR clone. Of the three R220 isolates without the S450L mutation, two had wild-type *rpoB *sequences and were RIF-susceptible, and the third had a distinct RIF-resistance mutation: H445Y.

**Figure 7:**
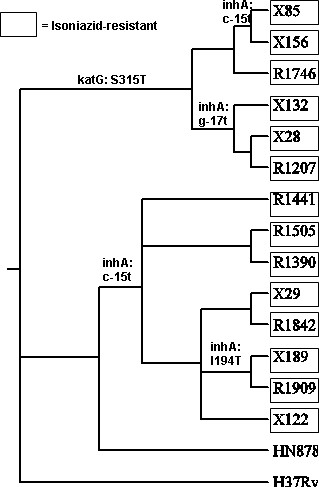
**Mutations in *inhA *and *katG *related to isoniazid resistance**. All strains except HN878 are isoniazid-resistant.

With respect to isoniazid resistance, Figure 7 shows that the c-15t *inhA *promoter mutation arose independently in both the R220 and R86 clusters. Whereas the c-15t mutation is found in all 8 strains of R220, it is only found in 2 of the 6 strains in the R85 cluster. However, the g-17t promoter mutation occurs in three other strains in the R86 cluster. The c-15t and g-17t *inhA *promoter mutations in the R86 cluster both occurred in the context of the *katG *S315T mutation, which occurs on an earlier branch and is found in all the strains of the R86 cluster. Simultaneous mutations in *katG *and the *inhA *promoter are frequently observed in clinical isolates [42], although it is unexpected that the *inhA *promoter mutations, which confer lower-level resistance to isoniazid, apparently occurred second. In contrast, the Ile194Thr mutation in *inhA *in isolates X189 and R1909 appears to have been acquired subsequent to the c-15t *inhA *promoter mutation common to the R220 strains, probably conferring higher-level resistance.

**Figure 8:**
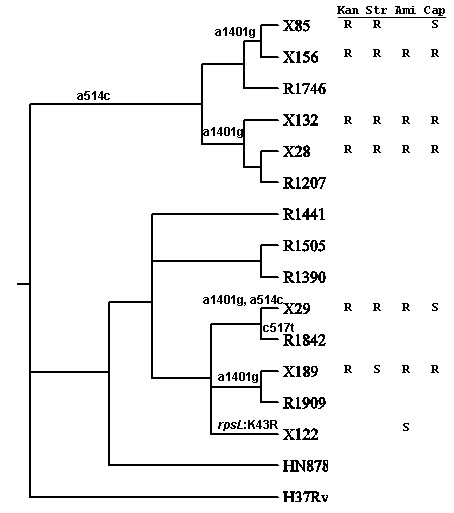
**Mutations in *rrs *and *rpsL *related to aminoglycoside resistance**. Kan = kanamycin, Ami = amikacin, Cap = capreomycin, Str = streptomycin. R = resistant. S = sensitive. Blank means not tested.

When the mutations in *rrs *and *rpsL *are mapped onto the phylogeny of the Beijing isolates (Figure 8), it is found that the R86 strains all shared mutation a516g in *rrs*, suggesting that streptomycin resistance arose in a common ancestor. The *rrs *a1401g mutation, responsible for kanamycin resistance, was observed in 5 of the 6 cluster R86 isolates, but not in isolate R1746. Therefore, it most likely has arisen twice within this cluster (unless there was a reversion to wild-type in R1746). In the R220 cluster, mutations (and most likely aminoglycoside resistance) were less prevalent. Isolate R1842 has a unique c517t *rrs *mutation that presumably also causes streptomycin resistance, and X122 is the only strain that has the K43R mutation in *rpsL*.

**Figure 9:**
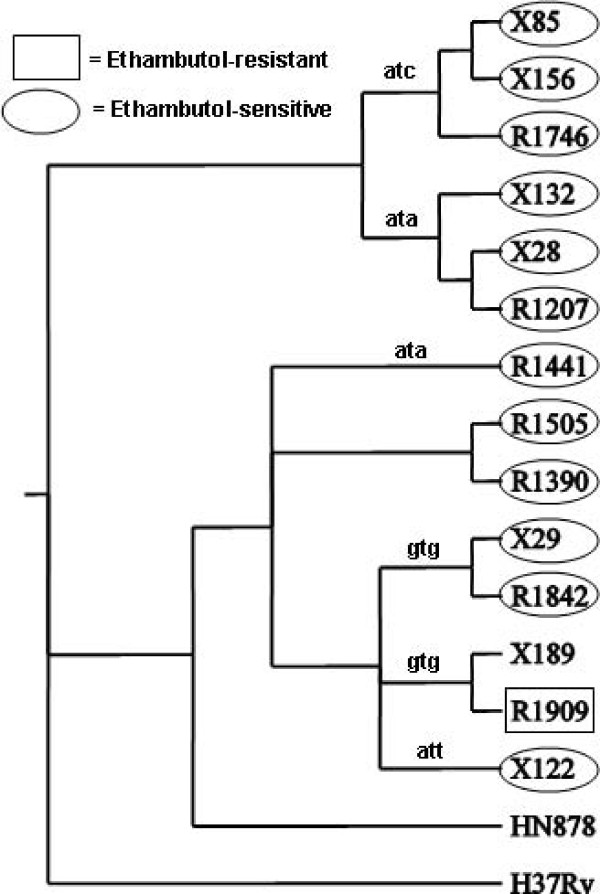
**Mutations in *embB *related to ethambutol resistance**. The mutations are shown as codons replacing Met306 (atg).

Finally, among the 14 Beijing isolates (both clusters), there appear to be six distinct mutations of Met306 in *embB *(Figure 9).

## Discussion

Our data suggests that extensive drug-resistance (beyond MDR) among Beijing strains in the Western Cape of South Africa is evolving and spreading adaptively rather than by clonal expansion. The phylogenetic analysis suggests that MDR drug resistance is being transmitted clonally, as demonstrated by the observation that isolates in the R220 cluster share the same c-15t *inhA *promoter mutation, and isolates in the R86 cluster share the same KatG S315T mutation. Similarly, many of the strains share the RpoB S450L mutation (though acquired separately in each cluster). This is consistent with the view that MDR-TB in the Western Cape is spreading by transmission [27]. However, mutations in other drug-resistance-related genes are highly homoplaseous, showing that the XDR mutations are not clonally related, even within these tightly defined clusters. For example, among the eight R220 strains sequenced, fluoroquinolone resistance was acquired three times independently, ethambutol resistance was acquired four times independently, and resistance to aminoglycosides was acquired four times independently.

The repeated acquisition of drug resistance mutations in these strains suggests that XDR isolates are less fit and do not propagate or compete well among the circulating population of Beijing variants. If there were one well-adapted XDR clone that had perhaps acquired compensatory mutations, then it would presumably result in identical drug-resistance mutations being transmitted through the population, which is not the case. This is consistent with the findings of van der Spuy [28], who found that, although Beijing strains as a whole were more prevalent and hence presumably more fit than other TB genotypes, drug resistant strains were not the predominant contributor to the rise of Beijing in the Cape Town region (but rather drug-susceptible isolates), probably due to increased fitness cost associated with drug-resistance mutations. Thus, although the Beijing strain as a whole appears to be more transmissible than other strains of TB, accounting for its success and world-wide dissemination, this does not appear to directly explain the spread of XDR drug-resistance among Beijing strains, as isolates in the region display varying drug-resistance mutations and hence do not represent the transmission of an identical clone. Diversity of resistance mutations among Beijing strains has also been observed in Japan [25] and Russia [22], again indicating independent acquisition.

This pattern mimics what has been observed for the LCC strains ('Low Copy Clade,' so-called because they have only 5 copies of the IS6110 insertion sequence), which are also prevalent in South Africa [59], although LCC strains are not of the Beijing genotype, but instead are in principle genetic group 2. Cluster analysis of drug resistance mutations in LCC isolates showed that they could not all be explained by a single sequence of acquisition, suggesting they arose multiple times independently [60]. However, previous studies have examined phylogenetic relationships using only a limited set of markers, including the drug-resistance mutations themselves, whereas our study is the first to use a genome-wide catalog of SNPs to assess relationships among strains independent of the drug-resistance mutations.

The repeated development of XDR drug resistance among Beijing strains of tuberculosis in the Western Cape region differs from other outbreaks of drug-resistant TB, such as in New York City in the 1990's [17,18], as well as in KwaZulu-Natal, South Africa more recently [61,62]. Each outbreak occurred in a defined geographical setting and was demonstrated to be due to transmission of clones with identical drug-resistance markers. In contrast, our findings suggest that XDR drug resistance in the Beijing strains in the Western Cape is not spreading clonally, but continues to be acquired independently in different strains. The drug-resistant TB outbreak in the Western Cape of South Africa covers a wider geographical region (spanning hundreds of miles). Thus the strains we analyzed are more distantly related, although they share a common ancestor, which is in turn reflected by the number of SNPs differentiating the respective isolates. The wide distribution of these strains demonstrates how failure to contain an initial drug resistant strain leads to both spread and acquisition of additional resistance markers under the right conditions, thereby emphasizing the need for rapid and accurate diagnosis of drug resistance.

There are a number of possible explanations for the increased association of drug resistance with the Beijing strain [34,63]. One hypothesis is that the Beijing strain is more adaptive, allowing it to acquire mutations more rapidly, which are then selected through the application of chemotherapy. It has been hypothesized that the apparent adaptiveness of the Beijing strain could be due to mutations in DNA repair genes *mutT2 *and *mutT4 *which might produce a hypermutator phenotype [64]. However, these mutations appear only in the most recent lineages, and are not found in the more ancestral lineages, including lineage 1, of which cluster R86 is a member. So it could not explain the extensive homoplasy of drug resistance among all these isolates. Furthermore, a comparison of the types of substitutions in the genomes of R220 isolates versus R86 isolates shows that the profiles are nearly identical (Figure 3), suggesting that there have not been functional changes to specific DNA repair genes or mechanisms, which might have been reflected in a bias of the substitution patterns [65,66]. No evidence has yet been found for hypermutation among clinical isolates, and the *in vitro rpoB *mutation rate (1.1 × 10^-8^) has been found to be in line with non-Beijing strains [67].

The primary limitation of our study is related to the selection of clinical isolates for sequencing. While all were of the Beijing genotype, the strains chosen for sequencing were selected to span a range of drug resistance from mono-resistant (all were resistant to INH) to XDR. While no sampling bias (in terms of age, gender, etc.) was intentionally applied, and the samples were isolated from patients in a variety of hospitals throughout the Western Cape region, it could be the case that our conclusions are specific to the small number of strains sequenced, or their shared resistance to isoniazid, and might not generalize to the broader TB epidemic within the region, or to outbreaks of drug-resistance and/or the Beijing strain of *M. tuberculosis *in other geographic locations around the world. Furthermore, no drug-susceptible strains from the Western Cape were sequenced, making the evolutionary relationships to the broader population of strains endemic to the region speculative. Finally, incidence of HIV is high the Western Cape region, and this could be an additional complicating factor that could influence the acquisition of drug resistance in these strains, as suggested in [68], but our study did not control for HIV status of the patients.

## Conclusions

Whole-genome sequencing and phylogenetic analysis of genetic differences among clinical isolates of the Beijing genotype from the Western Cape of South Africa suggests that, while MDR-TB is spreading by transmission in the region, additional drug resistance mutations are being acquired independently, and hence the spread of extensive drug resistance (XDR) appears to be non-clonal. The successful dissemination of these drug-resistant Beijing genotypes in South Africa should be a major concern for the National Tuberculosis Control Program, as the current strategy is unable to curb the spread of these strains [27]. Failure to contain (diagnose and treat) these strains has led to the evolution of XDR-TB through further acquisition of resistance markers.

## Authors' contributions

JS and PvH conceived of and designed the experiment. KD, TV, ES, RW, and NP collected the samples, performed genotyping and drug-susceptibility testing, and provided genomic DNA. XC ran the Illumina sequencer. YF and TI assembled the genome sequences and performed the analysis of the data. TI wrote the manuscript, and all authors contributed to editing it. All authors have read and approved the final manuscript.

## Supplementary Material

Additional file 1 **Supplementary details and comparative analysis of genome sequences of *M. tuberculosis *Beijing strains HN878, R1207 and X122**. This file contains a description of the polymorphisms observed among the genome sequences of *M. tuberculosis *Beijing strains HN878, R1207 and X122, including a catalog of SNPs, frameshift mutations, and coordinates of large-scale indels and IS6110 insertion sites. Click here for file
