# Supplementary material for: The non-clonality of drug resistance in Beijing-genotype isolates of Mycobacterium tuberculosis from the Western Cape of South Africa
Source: BMC Genomics. 2010 Nov 26;11:670. doi: 10.1186/1471-2164-11-670 (PMC3091785; doi:10.1186/1471-2164-11-670)
Supplement: Additional file 1 — Supplementary details and comparative analysis of genome sequences of M. tuberculosis Beijing strains HN878, R1207 and X122. This file contains a description of the polymorphisms observed among the genome sequences of M. tuberculosis Beijing strains HN878, R1207 and X122, including a catalog of SNPs, frameshift mutations, and coordinates of large-scale indels and IS6110 insertion sites. [file 1471-2164-11-670-S1.DOC]

**The non-clonality of drug resistance in Beijing-genotype isolates of *Mycobacterium tuberculosis* from the Western Cape of South Africa**

Thomas R. Ioerger, Yicheng Feng, Xiaohua Chen, Karen M. Dobos, Thomas C. Victor, Elizabeth M. Streicher, Robin M. Warren, Nicolaas C. Gey van Pittius Paul D. Van Helden, and James C. Sacchettini

### Additional File: Supplementary details and comparative analysis of genome sequences of *M. tuberculosis* Beijing strains HN878, R1207 and X122

### Sequence Analysis of HN878 Genome

In order to establish a complete genome sequence to use as a reference for sequencing of other members of the Beijing strain family, HN878 was chosen as a representative strain and the genome was sequenced using Solexa sequencing technology. HN878 was sequenced in paired-end mode using 36 bp reads. Mapping of reads to unique spacer sequences in the direct-repeats (DR) region confirms that HN878 has the characteristic 000000000003771 spoligotype associated with the Beijing strain family. The genome sequence of *M. tuberculosis* H37Rv (NCBI accession: NC_000962) was used as a reference sequence for comparative assembly of the HN878 genome. The mean depth of coverage was 70.8x, and 98.4% of the genome was covered by at least one read. The remaining uncovered regions are primarily associated with the family of PGRS genes, which have regions with 80-90% GC content, and are likely due to inefficient sequencing of GC-rich regions. For example, in HN878, 91.5% of sites with zero coverage (60,454/66,065) were located in PGRS genes.

Relative to the H37Rv genome, HN878 has 1546 single-nucleotide polymorphisms (SNPs) and 175 indels (insertion/deletions). We describe the small and large indels first. There are 75 genes that contain a stop-codon mutation or an indel in a coding region causing a frameshift, relative to the H37Rv reference sequence (listed in Table S1). Most of these cause truncations or small extensions of coding regions. Several frameshifts cause gene fusions, restoring functions ablated from H37Rv, including *pks15/1* (polyketide responsible for producing PGL; ), *aceAa* and *aceAb* (ICL2, putative dual-specificity isocitrate/methyl-isocitrate lyase ICL2; ), and *mmpL13a/b* (membrane lipid transporter) There are 11 large-scale indels with lengths over 1 kb, which are listed in Table S2. Several “insertions” represent parts of the *M. tuberculosis* genome that had been lost in H37Rv, but are present in other mycobacterial strains such as CDC1551. The 2.4 kb insertion at 3.8 Mb in HN878, containing a PPE protein and a transposase, appears to occur only in the F11 strain, and is not found in other mycobacteria. The 4.5 kb insertion at 2.22 Mb in HN878 appears to be completely novel; a BLAST search did not reveal similarity to any other DNA fragment/genome in the non-redundant database. GeneMark (http://exon.biology.gatech.edu) predicts that this region contains 7 open-reading frames of length 42 to 249 amino acids. There is also a deletion of 656 bp in HN878 that collapses *lppA/lppB*, which is a tandem duplication in H37Rv of a surface lipoprotein of unknown function .

There are 21 copies of the IS*6110* insertion element (transposon) in the HN878 genome. None of the insertion sites are shared identically with H37Rv, which has 16 copies (see Table S3). However, several of the insertion sites in HN878 are close to insertion sites in H37Rv, or in proximity to each other (these are indicated in Table S3). This observation supports the “hot-spot” theory , even-though no strong sequence-specific pattern has yet been identified. One particular 267-bp locus, called the *ipl* locus (for IS*6110*-insertion preference; ) has been investigated due to observation of multiple independent insertion events in different strains, including H37Rv (IS*6110* #1, at coordinate 888992), and HN878 has a separate insertion at this locus, 27 bp away. IS*6110* insertion elements are also associated with several large-scale insertion/deletions, including the loss of the 12kb Rv1754-Rv1765c gene cluster in HN878, and the “insertion” of the 5.1 kb *moaA* gene cluster, which was most likely lost in H37Rv due to an IS*6110*-mediated deletion (each region contains or is adjacent to an IS*6110*). An IS*6110* appears to be responsible for knocking out most of the direct-repeats region, as a copy is found at the end of the deleted region (in Rv2820). The genes disrupted by new insertions are shown in Table S3. The HN878 genome contains a characteristic copy inserted between *dnaA* and *dnaN* . However, it has only one copy in the NTF region, showing that it is modern but not of the W sublineage .

Among the 1546 SNPs, 1296 occur in protein-coding regions (CDS). Only 1063 of 3989 genes contain a polymorphism of any type, and the remaining 73% of the genes in the genome are identical with the sequence in H37Rv. No mutations typically associated with drug resistance were found in the following genes, consistent with the pan-susceptible phenotype of this clinical isolate: *inhA, katG, ethA, gyrA, iniABC, kasA, ndh, rpoB, rpsL, rrs, pncA,* and *embB*. HN878 has the *katG* R643L and *gyrA* S95T alleles, confirming its membership in principle genetic group 1 .

Non-synonymous SNPs are nearly twice as numerous as synonymous SNPs in the Beijing strains. Correcting for the total number of each type of site, the relative rate of non-synonymous to synonymous substitutions (Ka/Ks) between H37Rv and HN878 is calculated to be 0.557, using the method of Li, Wu, and Luo . The relative rate of non-synonymous to synonymous substitutions (Ka/Ks) between H37Rv and HN878, calculated to be 0.557, is high relative to other prokaryotes , but is in line with the Ka/Ks ratio found for other mycobacteria, which is estimated to be 0.57 , suggesting they are experiencing similar degrees of purifying selection.

**Table S1**. Mutations in HN878 affecting lengths of protein coding regions (changes in ORFs). Asterisk means stop codon. ‘/’ means multiple indels

|  | gene | frameshift  (bp) | length in  H37Rv (aa) | length in HN878b (aa) |
| --- | --- | --- | --- | --- |
| Rv0024 | *-* | -1 | 281 | 8 |
| Rv0045c | *-* | -2 | 298 | 216 |
| Rv0063 | *-* | +1/+1 | 479 | 191 |
| Rv0114 | *gmhB* | -1 | 190 | 223 |
| Rv0165c | *-* | -2 | 264 | 271 |
| Rv0197 | *-* | +2 | 762 | 748 |
| Rv0305c | *PPE6* | -1 | 963 | 3186 |
| Rv0354c | *PPE7* | +1 | 141 | 183 |
| Rv0388c | *PPE9* | +1/+1 | 180 | 443 |
| Rv0405 | *pks6* | -1 | 1402 | 31 |
| Rv0590 | *mce2B* | +1/+1 | 275 | 108 |
| Rv0907 | *-* | +1 | 532 | 44 |
| Rv1045 | *-* | -1 | 293 | 132 |
| Rv1046c | *-* | +1 | 174 | 197 |
| Rv1089 | *PE10* | -1 | 120 | 147 |
| Rv1128c | *-* | -1 | 451 | 318 |
| Rv1145 | *mmpL13a* | +1 | 303 | 781* |
| Rv1225c | *-* | +1 | 276 | 335 |
| Rv1258c | *-* | +1 | 419 | 231 |
| Rv1508c | *-* | -1/-8/-75 | 599 | 295 |
| Rv1549 | *fadD11.1* | +1 | 175 | 19 |
| Rv1551 | *plsB1* | -1 | 621 | 333 |
| Rv1668c | *-* | +10 | 372 | 591 |
| Rv1730c | *-* | -3/-19 | 517 | 447 |
| Rv1775 | *-* | -1 | 272 | 141 |
| Rv1888c | *-* | -10 | 186 | 239 |
| Rv1907c | *-* | -14 | 215 | 131 |
| Rv1915 | *aceAa* | +1 | 367 | 766* |
| Rv1997 | *ctpF* | -1 | 905 | 296 |
| Rv2027c | *-* | -1 | 573 | 270 |
| Rv2084 | *-* | +11 | 378 | 331 |
| Rv2148c | *-* | -1 | 258 | 259 |
| Rv2160A | *-* | +4 | 211 | 206 |
| Rv2160c | *-* | +4 | 113 | 458 |
| Rv2250A | *-* | -1 | 139 | 529 |
| Rv2251 | *-* | -1 | 475 | 94 |
| Rv2264c | *-* | +1/-18 | 592 | 585 |
| Rv2293c | *-* | +1 | 246 | 334 |
| Rv2339 | *mmpL9* | -1 | 962 | 95 |
| Rv2434c | *-* | -214 | 481 | 266 |
| Rv2437 | *-* | -1 | 139 | 73 |
| Rv2526 | *-* | -5 | 75 | 206 |
| Rv2541 | *-* | +1 | 135 | 18 |
| Rv2545 | *-* | -1 | 92 | 78 |
| Rv2879c | *-* | -1 | 189 | 99 |
| Rv2880c | *-* | -1 | 275 | 364 |
| Rv2947c | *pks15* | +7 | 496 | 2114* |
| Rv3203 | *lipV* | -1 | 224 | 43 |
| Rv3234c | *-* | +1 | 271 | 469 |
| Rv3293 | *pcd* | +35 | 494 | 24 |
| Rv3350c | *PPE56* | -1 | 3716 | 2066 |
| Rv3425 | *PPE57* | -1 | 176 | 86 |
| Rv3426 | *PPE58* | -1 | 232 | 176 |
| Rv3428c | *-* | -1 | 410 | 522 |
| Rv3453 | *-* | -1 | 110 | 561 |
| Rv3483c | *-* | +1 | 220 | 186 |
| Rv3510c | *-* | +1 | 278 | 246 |
| Rv3655c | *-* | -1 | 125 | 99 |
| Rv3725 | *-* | +1 | 309 | 333 |
| Rv3785 | *-* | -89 | 357 | 204 |
| Rv3829c | *-* | +1 | 536 | 515 |
| Rv3830c | *-* | +1 | 209 | 741 |
| Rv3847 | *-* | -1 | 177 | 208 |
| Rv3894c | *-* | -1 | 1396 | 541 |
| Rv3897c | *-* | +5 | 210 | 214 |
| Rv3911 | *sigM* | -1 | 222 | 196 |
| *genes with mutation in stop codon (ORF extension)* | | | | |
| Rv0325 | *-* | *75Q | 74 | 229 |
| Rv0836c | *-* | *218W | 217 | 240 |
| Rv1180 | *pks3* | *489Y | 488 | 2085 |
| Rv1783 | *-* | *436L | 435 | 1391 |
| Rv3898c | *-* | *111Q | 110 | 329 |
| *genes with non-sense mutation (ORF truncation)* | | | | |
| Rv0104 | *-* | Q380* | 504 | 379 |
| Rv0930 | *pstA1* | R305* | 308 | 304 |
| Rv2079 | *-* | Q609* | 656 | 608 |
| Rv2180c | *-* | W249* | 295 | 248 |
| Rv3303c | *lpdA* | C472* | 493 | 471 |
| Rv3367 | *PGRS51* | Y62* | 588 | 61 |
| Rv3872 | *PE35* | E99* | 99 | 98 |

* gene fusions: *mmpL13a* with *mmpL13b*, *aceAa* with *aceAb*, *pks15* with *pks1*

**Table S2**. Large-scale insertion/deletions in HN878 relative to H37Rv.

| **position*** | **type** | **size (bp)** | **genes disrupted/lost** | **genes inserted (or restored)** |
| --- | --- | --- | --- | --- |
| 79571 | del | -3466 | Rv0071-Rv0074 lost |  |
| 1332187 | del | -2848 | Rv1189/*sigI*-Rv1192 lost |  |
| 1480971 | ins | +1674 | Rv1319c disrupted | CDC1551:MT1360 (adenylate cyclase) |
| 1779281 | del | -9247 | Rv1572-Rv1587c |  |
| 1986639 | del | -11984 | Rv1754-Rv1765c lost; includes *plcD, cut1, wag22* |  |
| 2219418 | ins | +4495 | (in non-coding region) | unknown |
| 2268725 | ins | +5000 | Rv2024c disrupted | CDC1551:MT2082-MT-2086, includes helicase and cation efflux |
| 3119959 | del | -7973 | in Direct Repeats region |  |
| 3529067 | ins | +2150 | Rv3159c/PPE53 disrupted | CDC1551:MT3268-MT3270, hypothetical proteins |
| 3711736 | ins | +5157 | adjacent to IS*6110* | CDC1551:MT3426-MT3429, includes *moaA*, *afsR* transcription regulator |
| 3846841 | ins | +2422 | (in non-coding region) | F11:TBFG13461-13464, includes PPE, transposase |

*Coordinates are given relative to start position in H37Rv.

**Table S3**. IS*6110* insertion sites in HN878.

| **position in HN878** | **IS*6110* #** | **distance to closest IS*6110* in H37Rv** | **genes disrupted** |
| --- | --- | --- | --- |
| 1595 | HN:1 |  |  |
| 887044 | HN:2 | -27 bp upstream from H37Rv IS*6110* #1 | PPE16 |
| 1261010 | HN:3 |  | Rv1371 |
| 1540738 | HN:4 | +663 bp downstream from H37Rv IS*6110* #2 | *ctpD* (cation transporter ATPase) |
| 1655140 | HN:5 |  | Rv1754 |
| 1976622 | HN:6 | near H37Rv IS*6110* #3, loss of *plcD* region |  |
| 2018252 | HN:7 |  |  |
| 2144368 | HN:8 |  | PPE34 |
| 2249992 | HN:9 |  | Rv2016 |
| 2358269 | HN:10 | +122 bp downstream from H37Rv IS*6110* #5 |  |
| 2360410 | HN:11 | +911 bp downstream from H37Rv IS*6110* #5 | 31 bp upstream of PPE36 |
| 2624700 | HN:12 | -1529 bp upstream from H37Rv IS*6110* #8 | PPE38 |
| 3107428 | HN:13 |  | knock out DR region & Rv2818-Rv2820 |
| 3359183 | HN:14 |  |  |
| 3361015 | HN:15 | (474 bp apart from previous site) | between *esxS* & *esxR* (esat-6-like) |
| 3477244 | HN:16 |  | NTF region |
| 3534575 | HN:17 | (1854 bp apart from next site) |  |
| 3537788 | HN:18 | -2030 bp upstream from H37Rv IS*6110* #12 |  |
| 3701013 | HN:19 | +3571 bp downstream from H37Rv IS*6110* #14 | (part of 5kb insertion with *moaA*) |
| 3787332 | HN:20 | +1411 bp downstream from H37Rv IS*6110* #15 | *idsB* (polyprenyl synthetase) |
| 3835542 | HN:21 |  |  |

**Table S4**. IS*6110* insertion sites in X122, a representative of the R220 cluster. Shaded rows indicated differences from HN878.

| **site in HN878** | **site in X122** | **comments** |
| --- | --- | --- |
| 1595 | 1594 | same as HN:1 |
| 887044 | 887042 | same as HN:2 |
|  | 1024883 | **new** |
| 1261010 | 1261005 | same as HN:3 |
| 1540738 | 1540731 | same as HN:4 |
| 1655140 | 1655111 | same as HN:5 |
| 1976622 | 1985841 | same as HN:6 |
| 2018252 |  | HN:7 **deleted** |
| 2144368 |  | HN:8 **deleted** |
| 2249992 | 2259211 | same as HN:9 |
| 2358268 | 2367488 | same as HN:10 |
|  | 2369181 | **new** |
| 2360410 |  | HN:11 **deleted** |
| 2624700 | 2633919 | same as HN:12 |
| 3107528 | 3117301 | same as HN:13 |
| 3359183 | 3369052 | same as HN:14 |
| 3361015 | 3370884 | same as HN:15 |
| 3477244 | 3487112 | same as HN:16 |
| 3534575 |  | HN:17 **deleted** |
| 3537788 | 3547656 | same as HN:18 |
|  | 3706485 | **new** (disrupts *moaX*) |
| 3701013 | 3710881 | same as HN:19 |
|  | 3765676 | **new** (disrupts PPE56) |
| 3787332 | 3797200 | same as HN:20 |
| 3835542 | 3845410 | same as HN:21 |

### Genome Analysis of Beijing Strains in the R86 Cluster

The six R86 strains were all found to have the Beijing spoligotype and a common set of 14 IS*6110* insertion sites (Table S5) (although only 13 bands were observed in the RFLP fingerprints, Figure 1). However, only 6 of these sites were shared with HN878. The R86 strains all have an insertion in the *dnaA-dnaN* region, but they do not have any insertion in the NTF region (~3.48 Mb), classifying them as “ancestral” or “atypical” Beijing lineages . X132 has an additional unique insertion at 2.728 Mb (in PE25), though it is unclear why no extra band was observed in the RFLP pattern (Figure 1). Of the 8 newly-identified insertion sites, four are proximal to those in HN878 (151-1136 bp apart), supporting the notion of insertion hot spots , discussed below. The genes disrupted by these IS insertions include *fadD25* (fatty acid-CoA synthetase) and *lytB1* (penicillin tolerance protein), both putatively involved in cell-wall biosynthesis.

Using isolate R1207 as a representative of the R86 cluster, it is found to have the same large-scale insertion/deletions as HN878 compared to H37Rv, except that the 2.8kb region at 1.33Mb is not deleted in R1207. R1207 has the additional large indels listed in Table S6. There are 3 large insertions in PPE genes, loss of part of the *moaX* gene cluster inserted into HN878, and loss of Rv2016-Rv2019 (hypothetical proteins, plus a putative transcriptional regulator, Rv2017).

R86 strains are found to have a set of SNPs compared to H37Rv that only partially overlaps with the SNPs in HN878. Out of a selected set of 1885 SNPs, 1126 (59.7%) were found to be common among HN878 and all six R86 strains relative to H37Rv, HN878 has 297 (15.8%) unique SNPs, and the R86 strains have 256 (13.6%) shared SNPs not in HN878. Thus, while both HN878 and the R86 share many polymorphisms presumably acquired in a common ancestor of the Beijing family, they have each diverged significantly, reflecting the difference in their lineage memberships (sublineage 1 for R86 versus sublineage 6 for HN878).

### IS*6110* Transpositions

As has been suggested elsewhere , the IS*6110* insertion element appears to play a substantial role in the evolution of the *M. tuberculosis* genome. Between HN878 (21 copies) and H37Rv (16 copies), none are identical, and only 6 of 14 sites in HN878 are shared with the R86 cluster. Furthermore, the coding regions of numerous genes are disrupted (including several PPE genes; ), and multiple large-scale insertion/deletions are IS*6110*-associated. Even among the Beijing strains, there are substantial differences between the number of insertions and their chromosomal locations. While no specific sequence pattern has been identified for target insertion sites, several studies have proposed and presented evidence that IS*6110* insertions tend to cluster in certain hot spots . Our sequencing data supports this, with the co-occurrence of independent pairs of insertions at 11 sites separated by at most 663 bp (see Figure 2). This proximity is statistically unlikely, given that the average expected distance between IS*6110* elements is ~220 kb (20 insertions in 4.4Mb).

**Table S5**. Position of IS*6110* insertion sites in R86 strains. HN:x means the insertion site is the same as for site x in HN878, listed in Table S3.

| **coordinate**  **in R1207** | **description** | **genes disrupted** |
| --- | --- | --- |
| 1595 | same as HN:1 |  |
| 1023549 | 70 bp from site in X122 |  |
| 1541637 | near HN:4 (-540bp upstream) |  |
| 1711950 |  | *fadD25* |
| 1978160 | same as HN:6 (knock-out of *plcD* region) | Rv1754-Rv1765c |
| 2144226 | near HN:8 (-249bp upstream) | PPE34 |
| 2162834 |  | Rv1928c |
| 2251605 | near HN:9 (+151bp downstream) | Rv2016 |
| 2621769 | same as HN:12 | PPE38 |
| 2674185 |  | Rv2390c |
| 3108000 | HN:13 (end of direct repeats region) | Rv2820c |
| 3359757 | same as HN:14 |  |
| 3695834 | HN:19 truncated by 920bp; within *moaA* insert in HN878 |  |
| 3780140 | near HN:20 (-1136bp upstream) | *lytB1* |

**Table S6**. Large-scale insertion/deletions in R1207 relative to H37Rv, other than those shared with HN878 (Table S2).

| **position*** | **type** | **size (bp)** | **genes disrupted/lost** | **genes inserted (or restored)** |
| --- | --- | --- | --- | --- |
| 2263628 | del | -2540 | Rv2016-Rv2019 |  |
| 2634048 | ins | +2037 | PPE38 | tandem duplication of 1200 bp + 800 bp in between from H37Ra |
| 3501665 | ins | +1337 | PPE50 | part of PPE50 from BCG |
| 3711736 | del | -2291 | within *moaA* insert in HN878; deletes part of IS*6110* |  |
| 3731461 | ins | +704 | PPE54 | part of PPE54 from F11 |

*Coordinates are given relative to start position in H37Rv.

**Table S7**. SNPs associated with shorter branches in the phylogeny of the 15 Beijing strains. The coordinates are given relative to H37Rv. The nucleotide pattern shows the nucleotides present at each site for strains in the following order: H37Rv, HN878, **X122, R1909, R1842, R1390, X189, R1505, R1441, X29**, *R1207, X132, X28, R1746, X156, X85* (R220 isolates bold-faced, R86 italicized). ‘*’ indicates mutations known to be associated with drug resistance.

R86 cluster

SNPs unique to: X85 (1)

Rv0757 phoP 851982 GGGGGGGGGGGGGGGT T:[L125F]

SNPs unique to: X156 (6)

Rv0384c clpB 459759 GGGGGGGGGGGGGGCG C:[D748E]

Rv0663 atsD 756387 AAAAAAAAAAAAAACA C:[H84P]

Rv0758 phoR 853066 GGGGGGGGGGGGGGTG T:[G224V]

Rv1860 apa 2108465 GGGGGGGGGGGGGGTG T:[G244C]

Rv1872c lldD2 2122838 CCCCCCCCCCCCCCGC G:[G105A]

Rv3697c - 4139900 GGGGGGGGGGGGGGAG A:[L115F]

SNPs unique to: R1746 (12)

Rv0104 - 122503 CCCCCCCCCCCCCTCC T:[H63Y]

Rv0758 phoR 853668 CCCCCCCCCCCCCTCC T:[R425W]

non coding 886670 CCCCCCCCCCCCCACC

Rv0806c cpsY 900830 CCCCCCCCCCCCCACC A:[V167V]

Rv1527c pks5 1723402 AAAAAAAAAAAAAGAA G:[L1670L]

Rv1963c mce3R 2206637 CCCCCCCCCCCCCTCC T:[D56N]

Rv2041c - 2287684 CCCCCCCCCCCCCTCC T:[V55I]

Rv2364c era 2646501 AAAAAAAAAAAAAGAA G:[L58P]

Rv2484c - 2791907 AAAAAAAAAAAAACAA C:[I196M]

Rv2503c scoB 2818484 AAAAAAAAAAAAACAA C:[L215R]

Rv2963 - 3315785 GGGGGGGGGGGGGAGG A:[G184S]

Rv3795 embB 4247730 GGGGGGGGGGGGGAGG A:[G406D]

SNPs unique to: X132 (4)

Rv0393 - 474046 CCCCCCCCCCCTCCCC T:[V422V]

Rv0545c pitA 637420 GGGGGGGGGGGAGGGG A:[A15V]

Rv1203c - 1346553 CCCCCCCCCCCTCCCC

Rv2652c - 2976246 GGGGGGGGGGGAGGGG A:[V103V]

SNPs unique to: X28 (5)

Rv0469 umaA 560256 TTTTTTTTTTTTGTTT G:[D123E]

Rv0668 rpoC 766818 AAAAAAAAAAAACAAA C:[H1150P]

non coding 1531076 CCCCCCCCCCCCTCCC

Rv2082 - 2338741 GGGGGGGGGGGGAGGG A:[S11S]

Rv3870 - 4347144 TTTTTTTTTTTTGTTT G:[F222V]

SNPs shared among: X156 X85 (4)

Rv0342 iniA 410962 AAAAAAAAAAAAAAGG G:[H42R]

Rv1061 - 1184080 CCCCCCCCCCCCCCAA A:[N22K]

Rv2425c - 2722670 CCCCCCCCCCCCCCTT T:[E213E]

Rv3806c - 4269089 CCCCCCCCCCCCCCTT T:[A249T]

SNPs shared among: R1746 X156 X85 (5)

Rv0668 rpoC 764817 TTTTTTTTTTTTTGGG G:[V483G]

Rv0785 - 880387 CCCCCCCCCCCCCTTT T:[H350Y]

Rv2332 mez 2605150 GGGGGGGGGGGGGAAA A:[A15T]

non coding 3511368 GGGGGGGGGGGGGTTT

Rv2043c pncA 2289202 AAAAAA-AAAAAAGGG G:[C14R] *

SNPs shared among: R1207 X28 (4)

Rv0006 gyrA 7581 GGGGGGGGGGCGAGGG C:[D94H] *

Rv2930 fadD26 3244281 AAAAAAAAAAGAGAAA G:[T195T]

Rv3199c nudC 3571684 CCCCCCCCCCTCTCCC T:[S287N]

Rv3423c alr 3841083 AAAAAAAAAACACAAA C:[L113R]

SNPs shared among: R1207 X132 X28 (8)

Rv0642c mmaA4 737015 GGGGGGGGGGAAAGGG A:[D63D]

Rv0667 rpoB 761110 AAAAAAAAAATTTAAA T:[D435V] *

Rv0946c pgi 1055775 CCCCCCCCCCTTTCCC T:[R304H]

Rv1275 lprC 1424976 AAAAAAAAAAGGGAAA G:[T76A]

Rv1315 murA 1471474 GGGGGGGGGGAAAGGG A:[G385D]

non coding 1673423 GGGGGGGGGGTTTGGG

Rv2019 - 2266314 TTTTTTTTTTCCCTTT C:[I109T]

Rv2895c viuB 3204705 GGGGGGGGGGAAAGGG A:[G176G]

R220 cluster

SNPs unique to: R1441 (5)

Rv0267 narU 321866 AAAAAAAAGAAAAAAA G:[D179G]

non coding 934354 GGGGGGGGCGGGGGGG

Rv1565c - 1772339 CCCCCCCCTCCCCCCC T:[P497P]

non coding 3242010 TTTTTTTTCTTTTTTT

Rv3500c yrbE4B 3919989 TTTTTTTTCTTTTTTT C:[E25G]

SNPs unique to: R1505 (3)

Rv0667 rpoB 761139 CCCCCCCTCCCCCCCC T:[H445Y] *

Rv1364c - 1536355 GGGGGGGAGGGGGGGG A:[A430A]

Rv3911 sigM 4400736 CCCCCCCTCCCCCCCC T:[A184V]

SNPs unique to: R1390 (1)

Rv0245 - 296124 TTTTTCTTTTTTTTTT C:[S40S]

SNPs unique to: X29 (5)

Rv0758 phoR 852468 AAAAAAAAACAAAAAA C:[T25P]

Rv1358 - 1527645 CCCCCCCCCTCCCCCC T:[P345L]

Rv1908c katG 2155678 CCCCCCCCCTCCCCCC T:[R145H]

Rv3145 nuoA 3511919 TTTTTTTTTATTTTTT A:[F80I]

Rv3877 - 4355305 TTTTTTTTTCTTTTTT C:[V100A]

SNPs unique to: R1842 (10)

Rv0151c PE1 178431 AAAAGAAAAAAAAAAA G:[G293G]

Rv0166 fadD5 195309 TTTTGTTTTTTTTTTT G:[L106R]

Rv0668 rpoC 764841 TTTTCTTTTTTTTTTT C:[I491T]

Rv1028c kdpD 1150224 AAAAGAAAAAAAAAAA G:[V488A]

Rv1170 mshB 1300803 CCCCTCCCCCCCCCCC T:[T167I]

Rvnr01 rrs 1472362 CCCCTCCCCCCCCCCC c517t *

Rv1328 glgP 1494576 CCCCTCCCCCCCCCCC T:[R5C]

Rv1854c ndh 2102984 AAAAGAAAAAAAAAAA G:[I20T]

Rv2075c - 2331685 GGGGTGGGGGGGGGGG T:[R399S]

non coding 3893736 AAAAGAAAAAAAAAAA

SNPs unique to: X189 (3)

Rv0667 rpoB 762310 AAAAAAGAAAAAAAAA G:[H835R]

Rv1893 - 2140487 TTTTTTCTTTTTTTTT C:[M1T]

Rv2870c dxr 3182048 TTTTTTCTTTTTTTTT C:[M322V]

SNPs unique to: R1909 (3)

non coding 563435 AAAGAAAAAAAAAAAA

Rv0960 - 1073865 AAAGAAAAAAAAAAAA G:[T107T]

Rv2043c pncA 2289220 CCCTCCCCCCCCCCCC T:[D8N] *

SNPs unique to: X122 (8)

Rv0667 rpoB 761998 TTCTTTTTTTTTTTTT C:[L731P]

Rv0682 rpsL 781687 AAGAAAAAAAAAAAAA G:[K43R] *

Rv0873 fadE10 971512 TTGTTTTTTTTTTTTT G:[I336M]

non coding 2715342 CCGCCCCCCCCCCCCC

Rv3198c uvrD2 3569392 TTCTTTTTTTTTTTTT C:[N607S]

Rv3518c cyp142 3955273 AAGAAAAAAAAAAAAA G:[H83H]

Rv3854c ethA 4327247 GGTGGGGGGGGGGGGG T:[A76D]

Rv2043c pncA 2288933 GGCGGGGGGGGGGGGG C:[Y103*] *

SNPs shared among: R1390 R1505 (1)

Rv0062 celA1 65643 TTTTTGTGTTTTTTTT G:[L31W]

SNPs shared among: R1842 X29 (2)

Rv2571c - 2895305 TTTTGTTTTGTTTTTT G:[Q219P]

Rv3757c proW 4203252 GGGGAGGGGAGGGGGG A:[V16V]

SNPs shared among: R1909 X189 (2)

Rv1484 inhA 1674782 TTTCTTCTTTTTTTTT C:[I194T]

Rv3300c - 3685983 TTTCTTCTTTTTTTTT C:[*306W]

SNPs shared among: X122 R1909 R1842 X189 X29 (1)

Rv1564c treX 1769760 GGAAAGAGGAGGGGGG A:[A614A]

Mutations inconsistent with phylogeny:

SNPs shared among: X122 R1909 R1842 R1390 X189 R1505 R1441 X29 X156 X85 (1)

non coding 1673425 CCTTTTTTTTCCCCTT (inhA promoter) *

SNPs shared among: X29 R1207 X132 X28 R1746 X156 X85 (1)

Rvnr01 rrs 1472359 AAAAAAAAACCCCCCC a514c *

SNPs shared among: R1909 X189 X29 R1207 X132 X28 X156 X85 (1)

Rvnr01 rrs 1473246 AAAGAAGAAGGGGAGG a1401g *

SNPs shared among: X122 R1909 R1842 X189 R1746 X156 X85 (1)

Rv0667 rpoB 761155 CCTTTCTCCCCCCTTT T:[S450L] *

SNPs shared among: X122 R1441 R1207 X132 X28 R1746 X156 X85 (1)

Rv3795 embB 4247431 GGTGGGGGAGAAACCC T:[M306I] *

SNPs shared among: X122 R1842 X189 X29 X85 (1)

Rv0006 gyrA 7582 AAGACACAACAAAAAG G:[D94G] *

SNPs shared among: R1909 R1842 X189 X29 (1)

Rv3795 embB 4247429 AAAGGAGAAGAAAAAA G:[M306V] *

# References

1. Constant P, Perez E, Malaga W, Laneelle MA, Saurel O, Daffe M, Guilhot C: **Role of the pks15/1 gene in the biosynthesis of phenolglycolipids in the Mycobacterium tuberculosis complex. Evidence that all strains synthesize glycosylated p-hydroxybenzoic methyl esters and that strains devoid of phenolglycolipids harbor a frameshift mutation in the pks15/1 gene**. *J Biol Chem* 2002, **277**(41):38148-38158.

2. Upton AM, McKinney JD: **Role of the methylcitrate cycle in propionate metabolism and detoxification in Mycobacterium smegmatis**. *Microbiology* 2007, **153**(Pt 12):3973-3982.

3. Grana M, Bellinzoni M, Bellalou J, Haouz A, Miras I, Buschiazzo A, Winter N, Alzari PM: **Crystal structure of Mycobacterium tuberculosis LppA, a lipoprotein confined to pathogenic mycobacteria**. *Proteins* 2010, **78**(3):769-772.

4. Sampson SL, Richardson M, Van Helden PD, Warren RM: **IS6110-mediated deletion polymorphism in isogenic strains of Mycobacterium tuberculosis**. *J Clin Microbiol* 2004, **42**(2):895-898.

5. Fang Z, Forbes KJ: **A Mycobacterium tuberculosis IS6110 preferential locus (ipl) for insertion into the genome**. *J Clin Microbiol* 1997, **35**(2):479-481.

6. Turcios L, Casart Y, Florez I, de Waard J, Salazar L: **Characterization of IS6110 insertions in the dnaA-dnaN intergenic region of Mycobacterium tuberculosis clinical isolates**. *Clin Microbiol Infect* 2009, **15**(2):200-203.

7. Plikaytis BB, Marden JL, Crawford JT, Woodley CL, Butler WR, Shinnick TM: **Multiplex PCR assay specific for the multidrug-resistant strain W of Mycobacterium tuberculosis**. *J Clin Microbiol* 1994, **32**(6):1542-1546.

8. Kurepina NE, Sreevatsan S, Plikaytis BB, Bifani PJ, Connell ND, Donnelly RJ, van Sooligen D, Musser JM, Kreiswirth BN: **Characterization of the phylogenetic distribution and chromosomal insertion sites of five IS6110 elements in Mycobacterium tuberculosis: non-random integration in the dnaA-dnaN region**. *Tuber Lung Dis* 1998, **79**(1):31-42.

9. Mokrousov I, Ly HM, Otten T, Lan NN, Vyshnevskyi B, Hoffner S, Narvskaya O: **Origin and primary dispersal of the Mycobacterium tuberculosis Beijing genotype: clues from human phylogeography**. *Genome Res* 2005, **15**(10):1357-1364.

10. Sreevatsan S, Pan X, Stockbauer KE, Connell ND, Kreiswirth BN, Whittam TS, Musser JM: **Restricted structural gene polymorphism in the Mycobacterium tuberculosis complex indicates evolutionarily recent global dissemination**. *Proc Natl Acad Sci U S A* 1997, **94**(18):9869-9874.

11. Li WH, Wu CI, Luo CC: **A new method for estimating synonymous and nonsynonymous rates of nucleotide substitution considering the relative likelihood of nucleotide and codon changes**. *Mol Biol Evol* 1985, **2**(2):150-174.

12. Ochman H, Elwyn S, Moran NA: **Calibrating bacterial evolution**. *Proc Natl Acad Sci U S A* 1999, **96**(22):12638-12643.

13. Gutierrez MC, Brisse S, Brosch R, Fabre M, Omais B, Marmiesse M, Supply P, Vincent V: **Ancient origin and gene mosaicism of the progenitor of Mycobacterium tuberculosis**. *PLoS Pathog* 2005, **1**(1):e5.

14. Daubin V, Moran NA: **Comment on "The origins of genome complexity"**. *Science* 2004, **306**(5698):978; author reply 978.

15. Hershberg R, Lipatov M, Small PM, Sheffer H, Niemann S, Homolka S, Roach JC, Kremer K, Petrov DA, Feldman MW *et al*: **High functional diversity in Mycobacterium tuberculosis driven by genetic drift and human demography**. *PLoS Biol* 2008, **6**(12):e311.

16. Mokrousov I, Jiao WW, Sun GZ, Liu JW, Valcheva V, Li M, Narvskaya O, Shen AD: **Evolution of drug resistance in different sublineages of Mycobacterium tuberculosis Beijing genotype**. *Antimicrob Agents Chemother* 2006, **50**(8):2820-2823.

17. McEvoy CR, Falmer AA, Gey van Pittius NC, Victor TC, van Helden PD, Warren RM: **The role of IS6110 in the evolution of Mycobacterium tuberculosis**. *Tuberculosis (Edinb)* 2007, **87**(5):393-404.

18. McEvoy CR, van Helden PD, Warren RM, Gey van Pittius NC: **Evidence for a rapid rate of molecular evolution at the hypervariable and immunogenic Mycobacterium tuberculosis PPE38 gene region**. *BMC Evol Biol* 2009, **9**:237.

19. Warren RM, Sampson SL, Richardson M, Van Der Spuy GD, Lombard CJ, Victor TC, van Helden PD: **Mapping of IS6110 flanking regions in clinical isolates of Mycobacterium tuberculosis demonstrates genome plasticity**. *Mol Microbiol* 2000, **37**(6):1405-1416.

20. Vera-Cabrera L, Hernandez-Vera MA, Welsh O, Johnson WM, Castro-Garza J: **Phospholipase region of Mycobacterium tuberculosis is a preferential locus for IS6110 transposition**. *J Clin Microbiol* 2001, **39**(10):3499-3504.
